# Learning disability register enrolment in young people in England: a cohort study using primary care data

**DOI:** 10.3399/BJGP.2026.0160

**Published:** 2026-08-11

**Authors:** Rakhee Shah, Karen Horridge, Sonia Saxena, Dougal S Hargreaves, Irene Petersen

**Affiliations:** 1 Mohn Centre for Children’s Health and Wellbeing, Department of Epidemiology and Biostatistics, School of Public Health, Imperial College London, London, UK; 2 University of Sunderland, Sunderland, UK; 3 Department of Primary Care and Public Health, School of Public Health, Imperial College London, London, UK; 4 Department of Primary Care & Population Health, University College London, London, UK

**Keywords:** cohort study, health policy, learning disability, prevalence, primary health care, young people

## Abstract

**Background:**

Learning disability registers in UK primary care support proactive care, but under-identification remains substantial. The 2019 NHS Long Term Plan introduced targets to increase enrolment.

**Aim:**

To estimate prevalence and rates of new registration to learning disability registers among young people aged 14–24 years in England and examine changes following the introduction of national targets.

**Design and setting:**

Population-based open cohort study using routinely collected primary care data from general practices in England contributing to the Clinical Practice Research Datalink Aurum database, 2015–2023.

**Method:**

Young people aged 14–24 years who had been registered ≥6 months were included. Annual rates of new registration per 10 000 person-years at risk (PYAR) and recorded prevalence were calculated, stratified by age, sex, region, and year.

**Results:**

Between 2015 and 2023, 11 776 young people were first recorded on a learning disability register. Rates of new registration increased in 2020–2021 across ages and sexes, peaking at 15.8 per 10 000 PYAR in males aged 14–17 years and 8.4 per 10 000 in females before returning to pre-2019 levels by 2023. Recorded prevalence remained low overall (0.03–0.5%; 3.6–52.7 per 10 000) but increased among those aged 18–24 years, with eight-to-10-fold rises between 2015 and 2023. Prevalence was higher in males (rate ratios 1.7–1.8, 95% confidence interval = 1.6 to 1.9).

**Conclusion:**

National targets coincided with a temporary rise in enrolment, but identification remains below expected prevalence. Sustained policy focus and systematic identification and coding by practices are needed to ensure equitable access to preventive care during the transition to adulthood.

## How this fits

Young people with learning disabilities experience marked health inequalities and depend on inclusion on GP learning disability registers to access annual learning disability health checks. However, many remain unregistered and therefore invisible to primary care systems. Using national data (2015–2023) the study found that although register inclusion rose following policy initiatives such as the NHS Long Term Plan (2019) overall coverage remains far below expected prevalence, with persistent sex and regional inequalities. Practices should not assume eligible young people are captured on their registers and must take proactive steps to identify, code, and recall them to ensure equitable access to preventive care during transition to adulthood.

## Introduction

People with learning disabilities in England experience substantial inequalities in physical and mental health, resulting in markedly higher avoidable mortality.^1–3^ On average, males with a learning disability die 19 years earlier and females 23 years earlier than their peers without a learning disability.^3^ The COVID-19 pandemic highlighted and amplified these disparities, showing little progress in life expectancy over the past two decades.^4^

Intellectual disability is a neurodevelopmental condition characterised by significant limitations in both intellectual functioning (such as reasoning and problem-solving) and adaptive functioning (including communication, social participation, and independent living).^5,6^ It is typically indicated by an IQ of <70 alongside clinical assessment.^5,6^ Identification requires onset during the developmental period, evidence of clinically significant impairments confirmed through standardised and clinical evaluation, and the exclusion of alternative explanations. Severity is classified as mild, moderate, severe, or profound, based on adaptive functioning and associated support needs.^5,6^ In the UK, the term ‘learning disability’ is commonly used interchangeably with intellectual disability in clinical and policy contexts.

Health inequalities emerge early, with the transition from child to adult services identified as a period of heightened vulnerability.^1,7^ Young people with learning disabilities and their families face barriers to accessing services, inconsistent information across health settings, and staff who may lack experience or hold discriminatory attitudes.^2^ The Learning from Lives and Deaths of People with Learning Disabilities and Autistic People (LeDeR) programme reports disproportionately high rates of preventable deaths, driven by inadequate health and social support, including lack of reasonable adjustments and diagnostic overshadowing.^3^

Annual learning disability health checks (ALDHCs) are structured assessments that take place in primary care, and they were introduced in 2009 and extended in 2014 to young people aged ≥14 years.^8^ ALDHCs aim to identify health issues early, promote accessible care,^9,10^ reduce preventable admissions to hospital,^11^ and support continuity of care. Access to ALDHCs depends on inclusion on GP learning disability registers;^12^ yet only around 12% of eligible people are registered.^13^ This limited coverage represents a major barrier to preventive care. Estimates suggest global prevalence of learning disability is around 1%, falling to 0.5% among adults,^14^ consistent with English^15^ and Scottish primary care data.^16^ If true prevalence is closer to 2%, as suggested by community surveys,^17^ approximately three-quarters of individuals remain unrecognised in primary care. Variability in coding practices across general practices and local authority registers further contributes to this gap, particularly among those with mild to moderate disabilities, the so-called ‘hidden majority’.^12,18^ For young people, reliance on educational records, such as special educational needs status, means ongoing needs may be lost on reaching adulthood.^15,19,20^

This study investigated the prevalence of recorded learning disabilities among young people in England within the context of the GP learning disability register and the ALDHC scheme, a complex intervention involving multiple interdependent steps from identification to follow-up care.^21,22^ The study specifically focused on the critical problem of under-identification; how effectively GP learning disability registers capture young people during the transition to adult services, who remains unregistered, and how policy initiatives such as the NHS Long Term Plan (2019)^23^ influence identification. By addressing these gaps, this study provides essential evidence on barriers within current systems and informs strategies to improve equity, continuity of care, and health outcomes for young people with learning disabilities.

## Method

### Data source

This study used data from the Clinical Practice Research Datalink (CPRD) Aurum dataset,^24^ containing primary care records from England for individuals registered with a GP between 1 January 2015 and 31 December 2023. CPRD Aurum is a primary care database of anonymised medical records from general practice in England, covering approximately 20% of the UK population; patients are broadly representative of the English general population in terms of age, sex, and ethnicity and in terms of rural and urban distribution.^24,25^ Information is collected during routine GP consultations and includes coded data received from secondary care and community services.^24^

### Study population

#### Numerator

Young people aged 14–24 years who were registered with a CPRD-participating general practice between 1 January 2015 and 31 December 2023 and recorded as being on the learning disability register were included. The SNOMED CT code 416075005 identifies individuals ‘on the learning disability register’ and is used within the Quality and Outcomes Framework (QOF) to ascertain learning disability status^26^ to remunerate general practices for maintaining a learning disability register. This code was confirmed by GPs and practice managers as the standard for maintaining registers, supporting QOF renumeration, and inviting individuals for annual health checks. The ALDHC was only extended to young people in 2014, and this is why the study start date of 2015 was selected.

#### Denominator

The denominator includes all young people aged 14–24 years regardless of whether they were on the learning disability register or not in the study period. Individuals were only included in the analysis if they had been registered with the practice for a minimum of 6 months, and if data were present for the whole calendar year.

#### Justification for age range

The age range of 14–24 years was chosen to capture a key transition period from adolescence to early adulthood. Age 14 years marks the start of incentivised annual health checks for people with learning disabilities in UK primary care, which may influence identification and coding. The upper limit of 24 years reflects the extended transition to adult services, when delays in diagnosis, coding, and registration may still occur. Including this range allowed assessment of both timely and delayed identification across a clinically relevant transition period.

#### First record of learning disability

Learning disability is a lifelong developmental condition and does not remit once diagnosed. Following the 2014 extension of GP learning disability registers and ALDHCs to young people age ≥14 years, this study examined the first recorded inclusion on the register to assess trends in identification and the impact of national policies, including the NHS Long Term Plan (2019).^23^ The first recording of ‘on the learning disability register’ was classified as the index date. ‘Cases’ recorded within 6 months of practice registration were excluded as likely ‘prevalent cases’. Deaths and patients who had left the practices were accounted for in the denominator (follow-up time).

#### Prevalence of learning disability

The analysis on prevalence of learning disability included all young people aged 14–24 years who were first recorded as being on the learning disability register as the numerator, and these individuals were then also included in the numerator in the annual analysis going forward. The denominator included all young people aged 14–24 years registered with a general practice between 2015 and 2023. Deaths and patients who had left the practices were accounted for.

#### Statistical analysis

The overall rate of new registrations to the GP learning disability register was estimated per 10 000 person years at risk (PYAR) for each year between 2015 to 2023. This was determined by totalling the number of patients with a first record of being placed on the ‘learning disability register’ for each year between 2015 and 2023 and then dividing by the total person years of follow-up for all patient records for each year, during the study period. The rate of new registrations was determined by age category (14–17, 18–21, 22–24 years), gender, geographic region, and by calendar year by restricting the person years of follow-up to the respective category in question. Person time was measured from the latest of: the date of general practice registration or 6 months after the 1 January 2015 to the earliest of: date of first being placed ‘on the learning disability register’, date of death, date patient left the practice, or 31 December 2023. To investigate the impact of the NHS Long Term Plan (2019) on rates of new learning disability registrations in primary care records, 95% confidence intervals (CIs) for the rate of new registrations to the learning disability register were calculated between 2015 and 2023 to assess for significant changes following its introduction in 2019.

The overall prevalence of being on the learning disability register was calculated by dividing the total number of young people on the learning disability register by the total number of registered young people between 2015 and 2023, accounting for deaths and young people who had left practices. Crude prevalence by age, gender, and geographic region was also determined. Multivariable Poisson regression analysis was used to examine changes in the prevalence of learning disability and the effect of age, gender, geographic region, and calendar year by controlling for respective variables. Likelihood ratio tests were used to examine potential interaction between age, sex and calendar year. Significant interactions between age category and calendar year and sex and calendar year were present and therefore results have been stratified by sex and age category.

All statistical analyses was completed using Stata (version 18).

#### Missing data on Index of Multiple Deprivation

There was a high level of missing data for patient-level Index of Multiple Deprivation (IMD); patient-level IMD was only available for two-thirds of young people on the learning disability register, owing to estimates being taken from the 2019 census. The study was therefore unable to reliably undertake analysis based on deprivation.

#### Public and patient involvement

Eighteen young people aged 14–24 years with learning disabilities were involved during conceptualisation of this study in three workshops. Only 17% (3/18) of young people with learning disabilities that the authors of the current study engaged with knew about the learning disability register, highlighting a substantial gap in awareness of a key mechanism for accessing reasonable adjustments and annual health checks. This engagement took place before the study was designed and directly informed the research question, prompting us to investigate access to the learning disability register specifically among young people with learning disabilities.

## Results

### First record of learning disability and impact of the NHS Long Term Plan on registrations

In total, 11 776 young people aged 14–24 years had their first record ‘on the learning disability register’ between the 1 January 2015 and the 31 December 2023. Of the new registrations to the learning disability register, 66% (7772/11 776) were males and 34% (4004/11 776) were females.

Rates of new registration to the learning disability register varied by age, gender, and calendar year between 2015 and 2023 (Figure 1). Across all age groups, males consistently had higher registration rates than females (Figure 1). Registration rates increased across all age groups and both sexes during 2020–2021, peaking at 15.8 per 10 000 PYAR among males aged 14–17 years and 8.4 per 10 000 PYAR among females of the same age in 2021, before declining to approximately pre-2019 levels by 2023 (Figure 1, Supplementary Table S1). The most pronounced increases were observed among young people aged 14–17 years and 18–21 years, whereas those aged 22–24 years had the lowest new registration rates, a smaller peak during 2020–2021, and a more sustained reduction in subsequent years (Figure 1). Overall, the pattern indicates a temporary rise in new registrations around 2020–2021 across age groups, followed by a return to lower levels.

**Figure 1.**
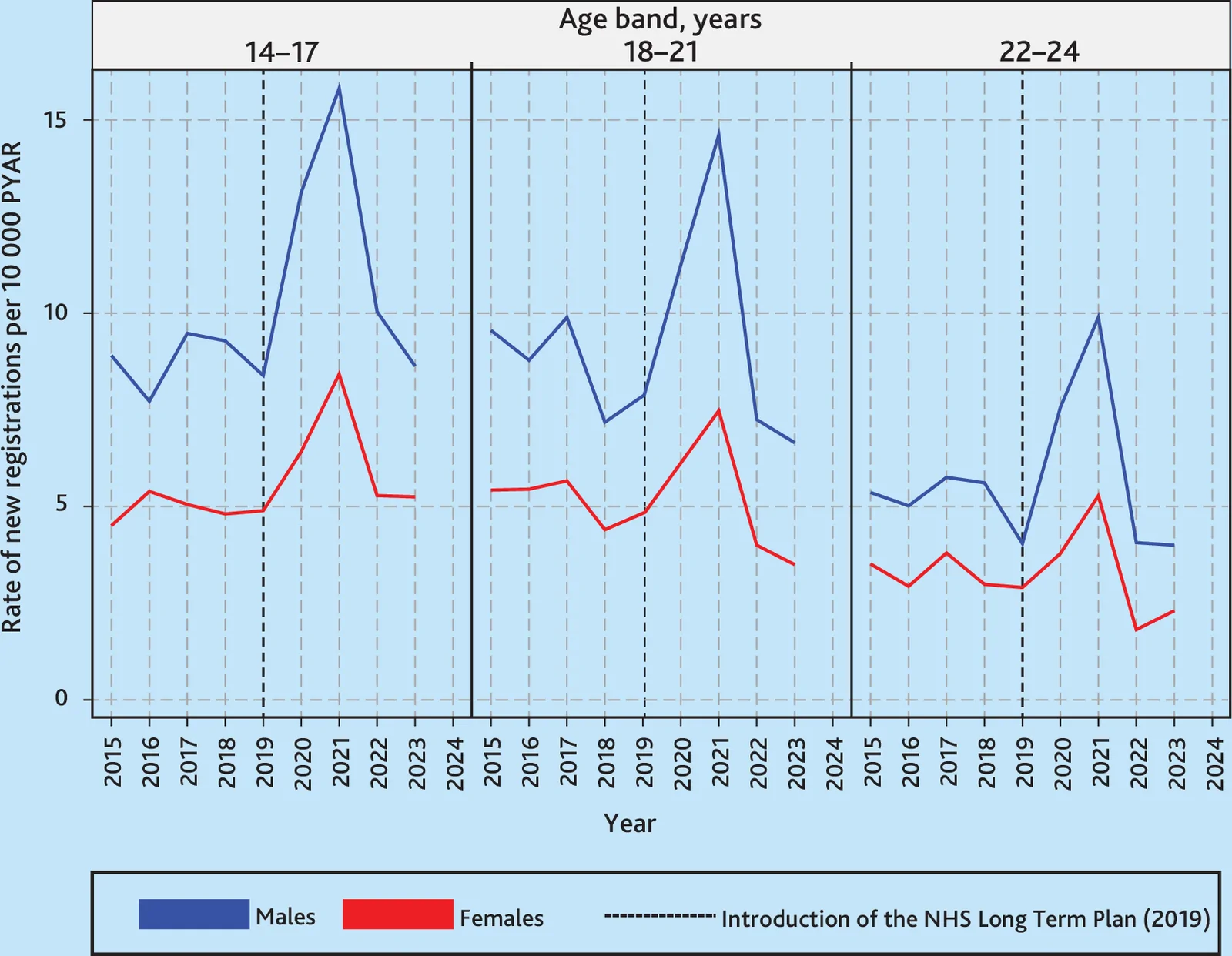
Rate of new registration to the learning disability register by gender and age category, 2015–2023, England. PYAR = person years at risk.

The temporal association between the introduction of the NHS Long Term Plan and rate of new registrations on the learning disability register differed across geographic regions (Figure 2, Supplementary Table S2). The increase in new registrations was more pronounced in London, the South East, West Midlands, and East of England in 2020 (Figure 2). There was an increase in new registrations among young males in the North East before the introduction of the NHS Long Term Plan (Figure 2).

**Figure 2.**
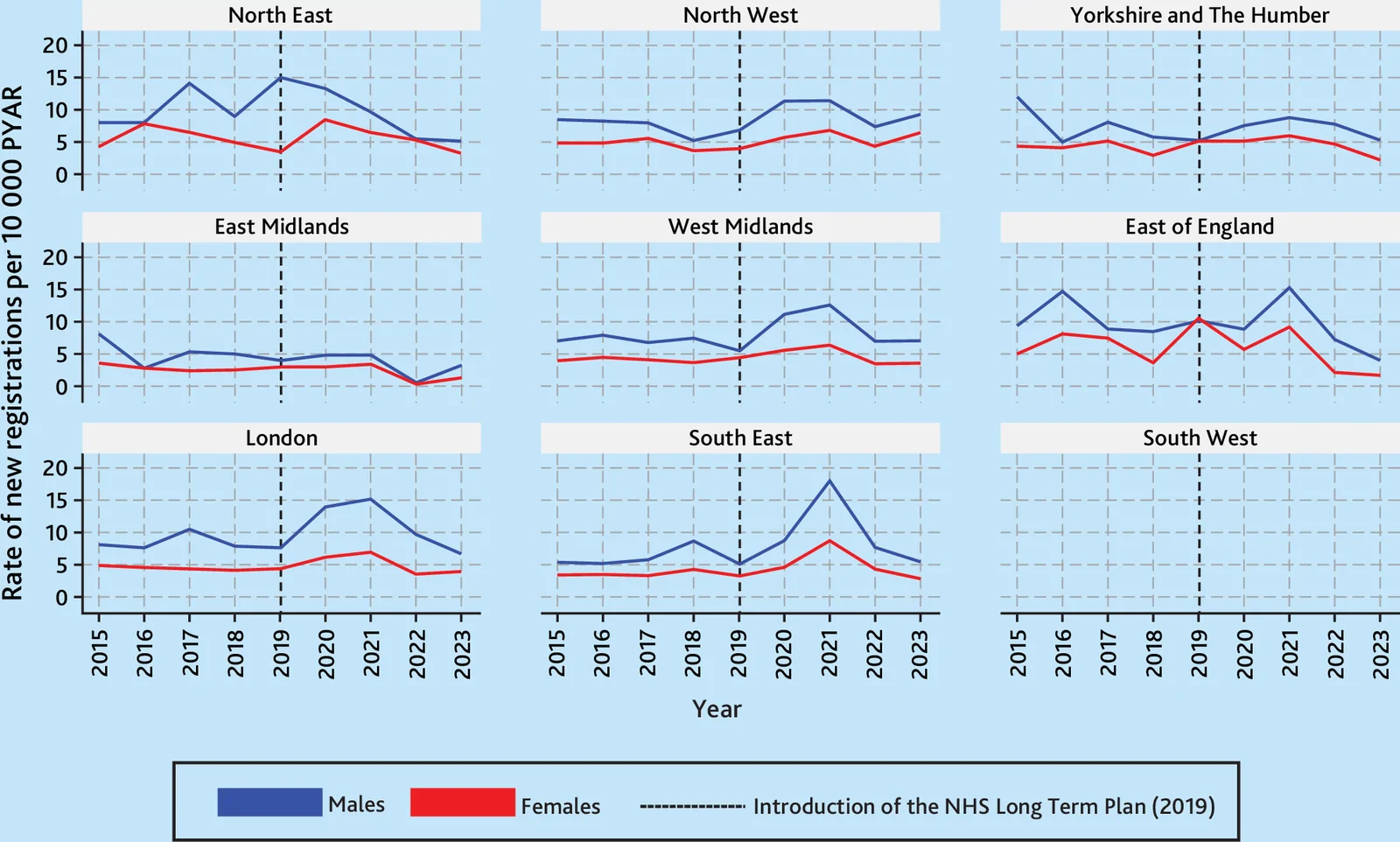
Rate of new registrations to the learning disability register for young people aged 14–24 years, By region, 2015–2023. PYAR = person years at risk.

### Prevalence

Recorded prevalence of learning disability increased substantially between 2015 and 2023 across all age groups and in both males and females (Table 1). The prevalence of young males aged 14–17 years with a learning disability on GP registers was 9.2 (95% CI = 8.1 to 10.4) per 10 000 in 2015 (Figure 3 and Table 1). This peaked at 29.5 (95% CI = 27.7 to 31.3) per 10 000 in 2021. The prevalence of learning disability for young females aged 14–17 years was 5.1 (95% CI = 4.2 to 6.1) per 10 000 in 2015 and peaked at 16.1 (95% CI = 14.7 to 17.5) per 10 000 females in 2021 (Table 1). Males aged 18–21 years had a prevalence of learning disability starting at 9.2 (95% CI = 8.1 to 10.3) per 10 000 males in 2015 but increased to 52.7 (95% CI = 50.3 to 55.1) per 10 000 in 2023. Females aged 18–21 years had a prevalence of learning disability of 5.0 (95% CI = 4.1 to 5.9) per 10 000 in 2015 and this peaked in 2022 at 31.2 (95% CI = 29.2 to 33.2) per 10 000. Males and females aged 22–24 years had slightly lower prevalence in 2023 compared with young people of the same gender than the 18–21 age group (Table 1).

**Figure 3.**
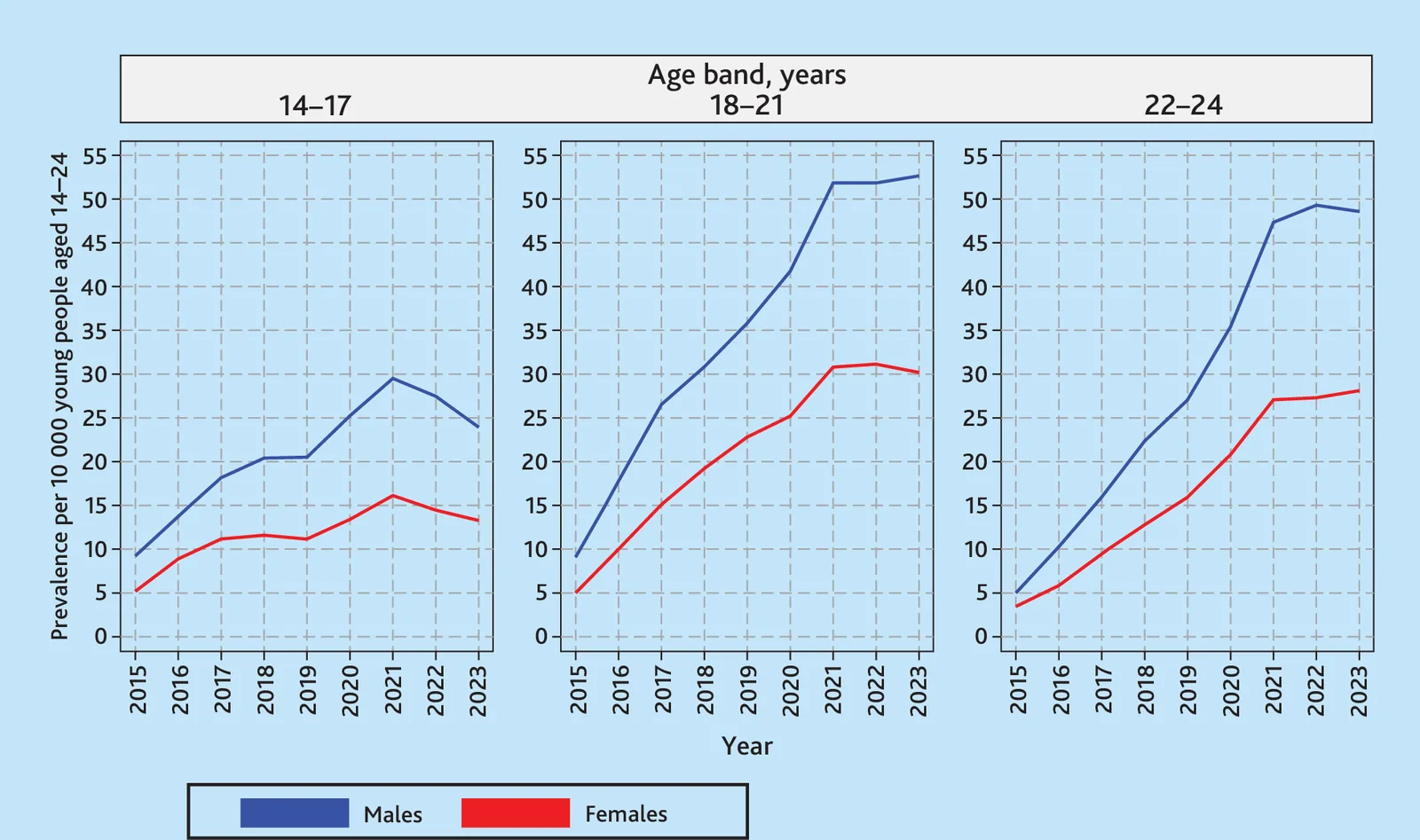
Administrative prevalence of young people aged 14–25 years on the learning disability register 2015–2023, in England.

**Table 1.** Administrative prevalence and prevalence rate ratio of young people aged 14–24 years on the learning disability register 2015–2023, in England

Age category and year	Prevalence per 10 000 males (95% CI)	Prevalence per 10 000 females (95% CI)	Prevalence rate ratio (2015 reference; adjusted for gender) (95% CI)
**14–17 years**			
2015	9.2 (8.1 to 10.4)	5.1 (4.2 to 6.1)	1.00
2016	13.8 (12.5 to 15.2)	8.9 (7.7 to 10.1)	1.6 (1.4 to 1.8)
2017	18.2 (16.7 to 19.9)	11.2 (9.9 to 12.6)	2.1 (1.8 to 2.3)
2018	20.4 (18.8 to 22.0)	11.6 (10.3 to 13.0)	2.2 (2.0 to 2.5)
2019	20.6 (19.1 to 22.2)	11.2 (10.0 to 12.5)	2.2 (2.0 to 2.5)
2020	25.3 (23.6 to 27.0)	13.4 (12.2 to 14.8)	2.7 (2.4 to 3.0)
2021	29.5 (27.7 to 31.3)	16.1 (14.7 to 17.5)	3.2 (2.8 to 3.6)
2022	27.5 (25.9 to 29.3)	14.5 (13.3 to 15.8)	3.0 (2.0 to 3.3)
2023	23.9 (22.4 to 25.5)	13.3 (12.2 to 14.6)	2.6 (2.3 to 2.9)
**18–21 years**			
2015	9.2 (8.1 to 10.3)	5.0 (4.1 to 5.9)	1.00
2016	17.9 (16.4 to 19.4)	10.1 (8.9 to 11.4)	2.0 (1.8 to 2.2)
2017	26.6 (24.8 to 28.5)	15.2 (13.7 to 16.7)	3.0 (2.6 to 3.3)
2018	30.1 (29.0 to 32.9)	19.3 (17.6 to 21.0)	3.5 (3.2 to 3.9)
2019	35.9 (33.9 to 38.1)	22.9 (21.1 to 24.7)	4.1 (3.7 to 4.6)
2020	41.8 (39.6 to 44.1)	25.2 (23.5 to 27.1)	4.7 (4.2 to 5.3)
2021	51.9 (49.4 to 54.4)	30.9 (28.9 to 32.9)	5.8 (5.2 to 6.5)
2022	51.9 (49.5 to 54.4)	31.2 (29.2 to 33.2)	5.9 (5.3 to 6.5)
2023	52.7 (50.3 to 55.1)	30.3 (28.4 to 32.2)	5.9 (5.3 to 6.5)
**22–24 years**			
2015	5.1 (4.3 to 6.1)	3.6 (2.8 to 4.4)	1.00
2016	10.4 (9.2 to 11.7)	6.0 (5.0 to 7.1)	1.9 (1.5 to 2.2)
2017	16.2 (14.6 to 17.8)	9.6 (8.3 to 10.9)	3.0 (2.5 to 3.5)
2018	22.4 (20.6 to 24.3)	13.0 (11.5 to 14.5)	4.1 (3.5 to 4.8)
2019	27.2 (25.3 to 29.3)	16.0 (14.4 to 17.7)	5.0 (4.3 to 5.8)
2020	35.5 (33.3 to 37.8)	20.9 (19.1 to 22.8)	6.5 (5.6 to 7.5)
2021	47.5 (45.0 to 50.2)	27.1 (25.1 to 29.2)	8.6 (7.5 to 10.0)
2022	49.3 (46.7 to 51.9)	27.2 (25.2 to 29.3)	8.9 (7.7 to 10.2)
2023	48.5 (46.0 to 51.1)	28.2 (26.2 to 30.3)	8.9 (7.7 to 10.2)

In general, the prevalence of being on the learning disability register was higher for males than females, thus, males were between 68% and 80% more likely than females to be on the register (prevalence rate ratio for males versus females aged 14–17 years: 1.8, 95% CI = 1.7 to 1.9, for those aged 18–21 years: 1.7, 95% CI = 1.6 to 1.7, and for those aged 22–24 years: 1.7, 95% CI = 1.7 to 1.8). The prevalence of learning disability for young females increased substantially over the time, among the oldest females (22–24 years old) there was an eight- to 10-fold increase in prevalence in the period 2015 to 2023 (Table 1). Similar increases were seen in males aged 22–24 years, prevalence increased almost 10-fold from 5.1 (95% CI = 4.3 to 6.1) per 10 000 in 2015 to 48.5 (95% CI =46.0 to 51.1) per 10 000 males in 2023.

These findings indicate a persistent gender disparity and a marked increase in the prevalence of learning disabilities between 2015 and 2023, particularly in young adults aged 18–24 years.

## Discussion

### Summary

This study provides, to the authors’ knowledge, the first national estimates of new registrations to learning disability registers among young people aged 14–24 years and the first analysis using CPRD primary care data to assess trends in relation to national policy, including the NHS Long Term Plan (2019).^23^ Young people aged 14–17 years had the highest rates of new registration compared with other age groups. Although inclusion on the learning disability register should occur at the point of identification and is not contingent on annual health checks, age 14 years represents an important clinical and operational threshold in UK primary care, marking increased structured review and transition-focused care, which may enhance case identification and recording. In addition, global developmental delay is typically recognised in early childhood,^27^ and most children with persistent developmental differences would expect to have had their learning disabilities formally identified and be included on the register by around age 14 years. Nonetheless, delays in formal identification and coding may occur in some cases, which could contribute to residual variation in registration patterns, particularly in younger age groups.

The lower rates of new registrations observed among those aged 22–24 years are likely explained by earlier identification and registration of most eligible individuals at younger ages, with relatively few first-time registrations occurring in the early 20s once diagnosis and transition into primary care systems have already taken place.

Rates of new registration and recorded prevalence increased markedly in 2020–2021 across all ages and sexes, with prevalence among 18–24-year-olds rising 10-fold. These changes coincided with the introduction of the NHS Long Term Plan (2019)^24^ and the COVID-19 pandemic, during which inclusion on learning disability registers facilitated access to priority vaccination and additional support.^28^ Although some studies suggest that there was substantial disruption to health and social care services for people with learning disabilities during the pandemic,^29^ these dynamics likely coexisted with increased identification in primary care, driven by heightened clinical awareness, changes in recording practices, and prioritisation of vulnerable groups during the pandemic.

Gains in new registrations were uneven, with substantial regional variation reflecting differences in local implementation. Without sustained national focus and explicit operational targets, inequalities in identification and access to preventive care during the transition to adulthood are likely to persist or widen for young people with learning disabilities. In addition, changes to UK primary care policy should be considered when interpreting these findings. Although maintenance of the learning disability register remains a contractual requirement since 2018 it is no longer directly linked to financial remuneration,^30^ which may affect the completeness and consistency of register recording across practices. As a result some of the observed changes in registration rates and prevalence may reflect variation in recording practices over time rather than true underlying changes in population need.

### Strengths and limitations

Strengths include the use of national CPRD primary care data, which are broadly representative of the English population and collected with consistent methods.^24^ This allowed robust estimation of both new registration rates and recorded prevalence over time, enabling meaningful comparisons across age groups and regions, unlike analyses based solely on absolute numbers.

A limitation of this study is the lack of ethnicity data for the denominator population, which precluded the calculation of ethnicity-adjusted rates of new registrations and prevalence. A further limitation of using primary care databases for secondary analysis is the potential under-ascertainment of individuals with learning disability, as identification relies on the accuracy and completeness of diagnostic coding in routine clinical practice. Previous studies have highlighted that learning disability is often underrecorded in primary care records, leading to incomplete case ascertainment and potential misclassification.^19^ Identification in secondary or community services may not always be communicated to general practice, which may also lead to lower case ascertainment in primary care.

### Comparison with existing literature

Recorded prevalence among young people aged 14–24 years (0.03–0.5%) in the current study remained far below population estimates of 2–5%,^17^ consistent with previous CPRD studies in adults and highlighting persistent under-identification in primary care.^31^ Existing literature suggests that under-identification is particularly pronounced among those with mild learning disabilities who do not receive formal educational or social care support, leaving them invisible in administrative datasets^19^ and limiting access to preventive services such as ALDHCs. Females were less likely than males to be recorded in the current study, which compares with existing literature^32^ reflecting both biological differences in neurodevelopmental risk and gendered referral patterns, with boys more often assessed for externalising behaviours.^33^

### Implications for practice

National targets for learning disability register enrolment and ALDHCs have driven short-term improvements in identification and care. However, the planned removal of these targets in the latest NHS operational guidance for 2025/2026 risks reversing recent gains.^34^ Although reinstating and sustaining operational targets may help maintain progress, targets alone are insufficient: a substantial number of young people with learning disabilities remain unrecorded and therefore miss out on preventive care.

General practices should adopt a proactive, systematic approach to case finding. This includes routine use of NHS England and NHS Improvement code lists,^35^ which identify clinical diagnoses that should automatically trigger inclusion on the learning disability register, as well as utilising a second group of conditions that may be associated with learning disability and therefore require individual clinical assessment to confirm eligibility. To support this process, GPs can use structured checklists such as the ‘Inclusion Tool’^35^ to help determine whether a patient should be added to the register. Practices should also regularly audit and review patients within these diagnostic groups to ensure accurate register status. Consistent use of standardised coding is essential to improve identification, ensure accurate recording of care processes, and support delivery of annual health checks and other preventive interventions.

Practices should consider having a named learning disability lead responsible for maintaining up-to-date knowledge and driving improvement in identification, register completeness, and care delivery.^36^ This role should act as a link with regional learning disability leads to share best practice and access relevant training opportunities, supporting continuous improvement at practice or primary care network level.

In conclusion, improving outcomes for young people with learning disabilities will require moving beyond reliance on targets alone towards proactive, structured identification embedded within routine primary care. Without consistent use of coding systems, practical case-finding tools, and designated clinical leadership, under-identification will persist. Sustained national targets alongside coordinated action across health and education are essential to ensure timely identification and equitable access to preventive care during the transition to adulthood.

## Data

The data that support the findings of this study are available from the Clinical Practice Research Datalink (CPRD) Aurum database. Access to CPRD Aurum data is subject to a licence agreement and approval by the CPRD Research Data Governance (RDG) process. Interested researchers may request access to the data from CPRD, subject to fulfilling the necessary ethical and governance requirements, including submission of a study protocol and approval of a data sharing agreement via the Independent Scientific Advisory Committee (ISAC) and the CPRD RDG process. The version of the CPRD Aurum dataset analysed in this study is CPRD Aurum June 2024.
